# OmpA, a Common Virulence Factor, Is Under RNA Thermometer Control in *Yersinia pseudotuberculosis*

**DOI:** 10.3389/fmicb.2021.687260

**Published:** 2021-05-17

**Authors:** Daniel Scheller, Christian Twittenhoff, Franziska Becker, Marcel Holler, Franz Narberhaus

**Affiliations:** Department of Microbial Biology, Ruhr University Bochum, Bochum, Germany

**Keywords:** gene expression, thermosensor, RNA structure, pathogen, virulence, outer membrane protein, porin

## Abstract

The outer membrane protein OmpA is a virulence factor in many mammalian pathogens. In previous global RNA structure probing studies, we found evidence for a temperature-modulated RNA structure in the 5'-untranslated region (5'-UTR) of the *Yersinia pseudotuberculosis ompA* transcript suggesting that opening of the structure at host-body temperature might relieve translational repression. Here, we support this hypothesis by quantitative reverse transcription PCR, translational reporter gene fusions, enzymatic RNA structure probing, and toeprinting assays. While *ompA* transcript levels decreased at 37°C compared to 25°C, translation of the transcript increased with increasing temperature. Biochemical experiments show that this is due to melting of the RNA structure, which permits ribosome binding to the 5'-UTR. A point mutation that locks the RNA structure in a closed conformation prevents translation by impairing ribosome access. Our findings add another common virulence factor to the growing list of pathogen-associated genes that are under RNA thermometer control.

## Introduction

OmpA is a highly abundant and conserved outer membrane protein in Gram-negative bacteria (Smith et al., 2007). It has a barrel-like structure that confers porin activity. Apart from its function in the influx and efflux of various compounds, OmpA is a multifaceted protein with various other functions, hence its designation as a molecular Swiss army knife. For instance, it plays an important role in envelope stability. In *Acinetobacter baumannii*, it was shown that two highly conserved residues anchor OmpA to the peptidoglycan layer (Park et al., 2012). In addition, OmpA forms complexes with the outer membrane protein Pal, which also associates with the peptidoglycan layer *via* a conserved α-helical interaction motif (Clavel et al., 1998).

OmpA and OmpA-like proteins have been recognized as virulence factors and are considered as potential vaccine candidates (Confer and Ayalew, 2013). The mode of action by which OmpA contributes to virulence ranges from adherence to epithelial cells and invasion (Smith et al., 2007; Gaddy et al., 2009) to biofilm formation (Weiser and Gotschlich, 1991; Azghani et al., 2002). In *Escherichia coli*, OmpA contributes to the resistance against serum killing by binding to the C4b-binding protein, which inhibits excessive activation of the complement system (Weiser and Gotschlich, 1991; Prasadarao et al., 2002). *A. baumannii* OmpA confers serum resistance through the acquisition of factor H to the cell surface (Kim et al., 2009). In addition, secreted *A. baumannii* OmpA is able to facilitate apoptosis by inducing mitochondrial damage and the release of proapoptotic molecules, leading to epithelial cell death (Choi et al., 2005). In a mouse model, *A. baumannii* OmpA is important to induce death as demonstrated by a killing defect of an *ompA* deletion strain (Sánchez-Encinales et al., 2017). In *E. coli* K1, OmpA is required to cause neonatal meningitis by enabling invasion of brain microvascular endothelial cells and crossing the blood-brain barrier (Prasadarao et al., 1996). OmpA in *Yersinia pestis* and *Yersinia pseudotuberculosis* is important for intracellular survival within macrophages as shown by reduced survival of strains lacking *ompA* (Bartra et al., 2012).

The amount of OmpA and other porins in the outer membrane is tightly regulated in response to numerous external conditions. Like many other genes coding for outer membrane proteins, *E. coli ompA* is subject to posttranscriptional regulation by small RNAs (sRNAs; Guillier et al., 2006). The paradigm of this type of control is the regulation of the *ompF* mRNA by the MicF sRNA (Mizuno et al., 1984). The 5'-untranslated region (5'-UTR) of *ompA* base-pairs with the sRNA MicA, which destabilizes the *ompA* transcript and requires the help of the RNA chaperone Hfq (Rasmussen et al., 2005; Udekwu et al., 2005). A second Hfq-dependent sRNA involved in *ompA* regulation in *E. coli* is RseX, which also regulates the expression of another outer membrane protein gene called *ompC* (Douchin et al., 2006).

A fundamentally different mode of riboregulation was recently reported for the *Shigella dysenteriae ompA* gene, which is regulated by a fourU-type RNA thermometer (RNAT; Murphy et al., 2020). *Shigella dysenteriae* is a serious foodborne pathogen causing the diarrheal disease shigellosis, and the OmpA protein is involved in intracellular spreading of the pathogen (Ambrosi et al., 2012). The last 20 residues of the 133-nucleotides long 5'-UTR of the *ompA* transcript fold into a simple hairpin structure, in which four uridines pair with GGAG, a part of the Shine-Dalgarno (SD) sequence. This structure is sufficiently strong to inhibit ribosome access at environmental temperatures but labile enough to denature at a host-body temperature in order to facilitate efficient translation resulting in an elevated level of the virulence factor (Murphy et al., 2020).

Regulating the production of virulence factors like OmpA in response to temperature is a sensible strategy since a shift to 37°C is among the most consistent changes upon arrival in the warm-blooded host. Therefore, many pathogens have established various mechanisms to measure the ambient temperature at the DNA, RNA, or protein level (Steinmann and Dersch, 2013; Mandin and Johansson, 2020). A widely used strategy in numerous pathogenic bacteria is the translational control of virulence-associated genes by RNATs (Loh et al., 2018). The sequence and structure of these regulatory elements are surprisingly diverse. Even orthologous genes in related organisms are controlled by unrelated RNATs, and this is also the case for the *ompA* gene. While the *Shigella ompA* transcript is regulated by a fourU thermometer (Murphy et al., 2020), the RNAT upstream of *ompA* in *Y. pseudotuberculosis* presented here is structurally disparate but functionally equivalent. Like *Shigella*, *Y. pseudotuberculosis* is a notorious foodborne pathogen causing gut-associated diseases. A considerable number of *Yersinia* virulence genes are temperature regulated, often by riboregulatory processes (Knittel et al., 2018). We identified a very promising RNAT upstream of *ompA* by a global RNA structuromics approach, in which *Y. pseudotuberculosis* RNA structures were probed at three different temperatures (Righetti et al., 2016). Here, we set out to study the structure-function relationship of this temperature-labile element and show that it is a functional RNAT allowing induction of OmpA levels at increasing temperature.

## Materials and Methods

### Bacterial Strains and Plasmids

Bacterial strains used in this study are listed in Table 1. Cells were grown in lysogeny broth (LB; 1% NaCl, 1% tryptone, and 0.5% yeast extract) at indicated temperatures. Cultures were supplemented with 150 μg/ml ampicillin when necessary. For the induction of the P_BAD_ promoter, the medium was supplemented with L-arabinose to a final concentration of 0.01% (*E. coli*) or 0.1% (*Y. pseudotuberculosis*), respectively.

**Table 1:** Bacterial strains.

Strain	Relevant characteristics	Reference
*Yersinia pseudotuberculosis* YPIII	pIB1, wild type	Bölin et al., 1982
*Escherichia coli* DH5α	*supE44*, Δ*lacU169* (ψ80*lacZ*Δ*M15*), *hsdR17*, *recA1*, *gyrA96*, *thi1*, and *relA1*	Hanahan, 1983

### Plasmid Construction

All utilized oligonucleotides and plasmids are summarized in Tables 2 and 3, respectively. Point mutations were generated by site-directed mutagenesis according to the instruction manual of the QuikChange^®^ mutagenesis kit (Agilent Technologies).

**Table 2:** Oligonucleotide list.

Name	Purpose	Plasmid	Sequence (5' → 3')
ompA_UTR_fw	Forward primer to amplify the 5' UTR of YPK_2630 (*ompA*) plus 30 bp of *ompA* coding region (−62 to +30 bp from ATG)	pBO4435	TTGCTAGCATTTTAACCAAGGGCTTAGC
ompA_UTR_rev	Reverse primer to amplify the 5' UTR of YPK_2630 (*ompA*) plus 30 bp of *ompA* coding region (−62 to +30 bp from ATG)	pBO4435	TTGAATTCCACTGCTAATGCGATAGCT
ompA_rep_fw	Mutagenesis forward primer to introduce the mutation U38UC into YPK_2630 (*ompA*) 5' UTR	pBO4451 pBO4921	GCTTTTAAAGCTCATTGCCTCATTTGGATGATAATGAGG
ompA_rep_rev	Mutagenesis forward primer to introduce the mutation U38UC into YPK_2630 (*ompA*) 5' UTR	pBO4451 pBO4921	CCTCATTATCATCCAAATGAGGCAATGAGCTTTAAAAGC
ompA_ro_fw	Forward primer to amplify YPK_2630 (*ompA*) 5' UTR with a T7 promoter for the construction of the runoff plasmids	pBO4920	AGAAATTAATACGACTCACTATAGGGATTTTAACCAAGGGCTTAGC
ompA_ro_rev	YPK_2630 (*ompA*) 5' UTR +30 bp from ATG; reverse primer with EcoRV site for the construction of the runoff plasmid for structure probing and primer extension inhibition	pBO4920	AAGATATCCACTGCTAATGCGATAGCT
RT_ompA_fw	Forward primer for detection of YPK_2630 (*ompA*) by qRT-PCR	–	CTGTAGTTGTTCTGGGCTTTGCTGAC
RT_ompA_rev	Reverse primer for detection of YPK_2630 (*ompA*) by qRT-PCR	–	CTTTAGAAACCAGGTAGTCACGCACG
RT_bgaB_fw	Forward primer for detection of *bgaB* by qRT-PCR	–	GACTGCAACTACTCCAGCTTGGTTTG
RT_bgaB_rev	Reverse primer for detection of *bgaB* by qRT-PCR	–	CTACTGCCAAACGAGAGAATGACACC
RT_nuoB_fw	Forward primer for detection of YPK_1561 (*nuoB*) by qRT-PCR	–	GATCCTCTCGAGCAACATG
RT_nuoB_rev	Reverse primer for detection of YPK_1561 (*nuoB*) by qRT-PCR	–	TAAAGCAGGTTCCGGCCA
RT_gyrB_fw	Forward primer for detection of YPK_0004 (*gyrB*) by qRT-PCR	–	TCGCCGTGAAGGTAAAGTTC
RT_gyrB_rev	Reverse primer for detection of YPK_0004 (*gyrB*) by qRT-PCR	–	CGTAATGGAAGTGGTCTTCT

**Table 3:** Plasmid list.

Plasmid	Relevant characteristics	Reference
pUC18	Cloning vector; Ap^r^	Yanisch-Perron et al., 1985
pBAD2-*bgaB*-His	*bgaB* reporter gene vector, Ap^r^, araC, P_BAD_ promoter, His-Tag at the C-terminal of BgaB	Righetti et al., 2016
pBO3146	pBAD2-*bgaB*-His; ICR between pYV0075(*yscW*) and pYV0076(*lcrF*) plus 9 bp of *lcrF* coding region (123 to +9 bp from lcrF ATG)	Righetti et al., 2016
pBO4435	pBAD2-*bgaB*-His; 5' UTR of YPK_2630 (*ompA*) plus 30 bp of *ompA* coding region (−62 to +30 bp from *ompA* ATG)	This study
pBO4451	pBAD2-*bgaB*-His; 5' UTR of YPK_2630 (*ompA*) plus 30 bp of *cnfY* coding region (−62 to +30 bp from *ompA* ATG), mutant Rep U38UC	This study
pBO4920	pUC18; YPK_2630 (*ompA*) 5' UTR plus coding region (−62 to +30 bp from *ompA* ATG); runoff plasmid for structure probing and primer extension inhibition	This study
pBO4921	pUC18; YPK_2630 (*ompA*) 5' UTR plus coding region (−62 to +30 bp from *ompA* ATG), mutant Rep U38UC; runoff plasmid for structure probing and primer extension inhibition	This study

The *ompA* RNAT-*bgaB* fusion plasmid (pBO4435) was constructed by first amplifying the YPK_2630 (*ompA*) 5'-UTR including 30 bp of the *ompA* coding region (108 bp) with primer pairs ompA_UTR_fw/ompA_UTR_rev, digested with NheI and EcoRI and ligated into pBAD2-*bgaB*-His. The repressed *ompA* RNAT-*bgaB* (U38UC) fusion was constructed by site-directed mutagenesis with primer pair ompA_rep_fw/ompA_rep_rev using pBO4435 as a template.

The runoff plasmid for *in vitro* transcription of the *ompA* RNAT (pBO4920) was constructed by blunt-end ligation of a PCR-amplified DNA fragment (primer pair ompA_ro_fw/ompA_ro_rev), comprising the T7 RNA polymerase promoter, and the *ompA* RNAT including 30 bp of the *ompA* coding region, into the EcoRV restriction site of pUC18. Insertion of the repressive mutation (U38UC) into pBO4920 was achieved by site-directed mutagenesis (primer pair ompA_rep_fw/ompA_rep_rev).

### Reporter Gene Activity Assay

*Escherichia coli* DH5α or *Y. pseudotuberculosis* YPIII cells carrying the *ompA* RNAT-*bgaB* fusion plasmids were grown overnight in LB supplemented with ampicillin at 25°C. Before being inoculated with an overnight culture to an optical density at 600 nm (OD_600_) of 0.1, LB media supplemented with ampicillin were pre-warmed to 25°C. After growth to an OD_600_ of 0.5, transcription was induced with 0.01% (*E. coli*) or 0.1% for (*Y. pseudotuberculosis*) L-arabinose, respectively. The culture was split and shifted to pre-warmed 100 ml flasks. After incubation for 30 min, 400 μl samples was subsequently taken for β-galactosidase assay, 2 ml samples for Western blotting, and 4 ml samples for RNA isolation. The β-galactosidase assay was carried out as described previously (Gaubig et al., 2011; Righetti et al., 2016).

### Western Blot Analysis

Cell pellets were resuspended in 1 × SDS sample buffer (2% SDS, 0.1% bromophenol blue, 1% 2-mercaptoethanol, 25% glycerol, 50 mM Tris/HCl, and pH 6.8) according to their optical density (100 μl per OD_600_ of 1). After boiling for 10 min at 95°C, samples were centrifugated (10 min, 13,000 rpm) and the supernatant was separated by SDS gel electrophoresis in 5% stacking and 12% separating gels. Size-separated proteins were transferred by tank blotting onto a nitrocellulose membrane (Hybond-C Extra, GE Healthcare). An anti-His-HRP conjugate antibody (Bio-Rad) was used in a 1:4,000 dilution. Luminescence signals were detected by incubating membranes with Immobilon Forte Western HRP substrate (Millipore) and the FluorChem SP (Alpha Innotec).

### RNA Extraction and Quantitative Reverse Transcription PCR

Total RNA was extracted using the peqGOLD TriFast reagent according to the manufacturer’s protocol. RNA samples were treated with Turbo^™^ DNase (TURBO DNA-free^™^ Kit, Invitrogen) to remove DNA contamination. cDNA synthesis was performed using the iScript^™^ cDNA synthesis Kit (Bio-Rad) according to the manufacturer’s protocol with 1 μg RNA per reaction. 2 μl of 1:10 diluted cDNA was mixed with 250 nM of each primer, 5 μl of 2× iTaq Universal SYBR Green Supermix, and 2.5 μl sterile water (Carl Roth). Amplification and detection were performed in a CFX Connect^™^ Real-Time System (Bio-Rad). Standard curves were used to calculate primer efficiency and determine the linear range of amplification. Relative transcript amounts were calculated using the primer efficiency corrected method (Pfaffl, 2001). The non-thermoregulated reference genes *gyrB* and *nuoB* were used for normalization.

### *In vitro* Transcription

RNA for RNA structure probing and primer extension inhibition experiments were synthesized *in vitro* by runoff transcription with T7 RNA polymerase (Thermo Scientific) from EcoRV-linearized plasmids (listed in Table 3) as previously described (Righetti et al., 2016).

### Enzymatic RNA Structure Probing

RNA structure probing of the 5'-UTR and 30 nt of *ompA* was performed as described previously (^43,44^). Briefly, *in vitro* transcribed and 5'-[^32^P]-labeled RNA (3,000 cpm) was mixed with buffer and tRNAs, preincubated for 5 min at the respective temperature, and treated with T1 (0.0017U; Invitrogen) or T2 (0.056U; MoBiTec) RNases for 5 min. For digestion with RNase T1 and RNase T2, a 5 × TN buffer (100 mM Tris acetate, pH 7, and 500 mM NaCl) was used. An alkaline hydrolysis ladder was prepared as described in Brantl and Wagner (1994), meaning 60,000 cpm of labeled RNA was mixed with tRNAs and ladder buffer (1M Na_2_CO_3_ and 1M NaHCO_3_; pH 9) incubated for 2 min at 90°C. The T1 ladder was generated by using 30,000 cpm-labeled RNA. The samples were heated with 2 μl sequencing buffer (provided with RNase T1) at 90°C. Afterward, the RNA was incubated with the T1 RNase for 5 min at 37°C. All reactions were stopped by the addition of formamide loading dye and denaturation at 95°C.

### Primer Extension Inhibition Analysis (Toeprinting)

Toeprinting analysis was performed with 30S ribosomal subunits, *in vitro* transcribed RNA, and tRNA^fMet^ (Sigma-Aldrich) according to a protocol as described before (Hartz et al., 1988). The 5'-[^32^P]-labeled oligonucleotide ompA_ro_rv, complementary to the 3' end of the *ompA* mRNA, was used as a primer for cDNA synthesis. The radiolabeled primer (0.16 pmol) was annealed to the *ompA* mRNA (0.08 pmol) and incubated with 30S ribosomal subunits (24 pmol) or Watanabe buffer [60 mM HEPES/KOH, 10.5 mM Mg(CH_3_COO)_2_, 690 mM NH_4_COO, 12 mM 2-mercaptoethanol, 10 mM spermidine, and 0.25 mM spermine] in the presence of tRNA^fMet^ (8 pmol) at 25, 37, or 42°C for 10 min. After the addition of 2 μl MMLV-Mix [VD+Mg^2+^ buffer, BSA, dNTPs, and 800 U MMLV reverse transcriptase (Invitrogen)], cDNA synthesis was performed for 10 min at 37°C. The reaction was stopped by the addition of formamide loading dye, and the samples were separated on an 8% denaturing polyacrylamide gel. The Thermo Sequenase Cycle Sequencing Kit (Applied Biosystems) was used for sequencing reactions with plasmid pBO4920 as a template and radiolabeled primer ompA_ro_rv.

## Results

### The *ompA* 5'-UTR Contains a Thermoresponsive RNA Structure, Which Facilitates Temperature-Dependent Regulation

**Figure 1:**
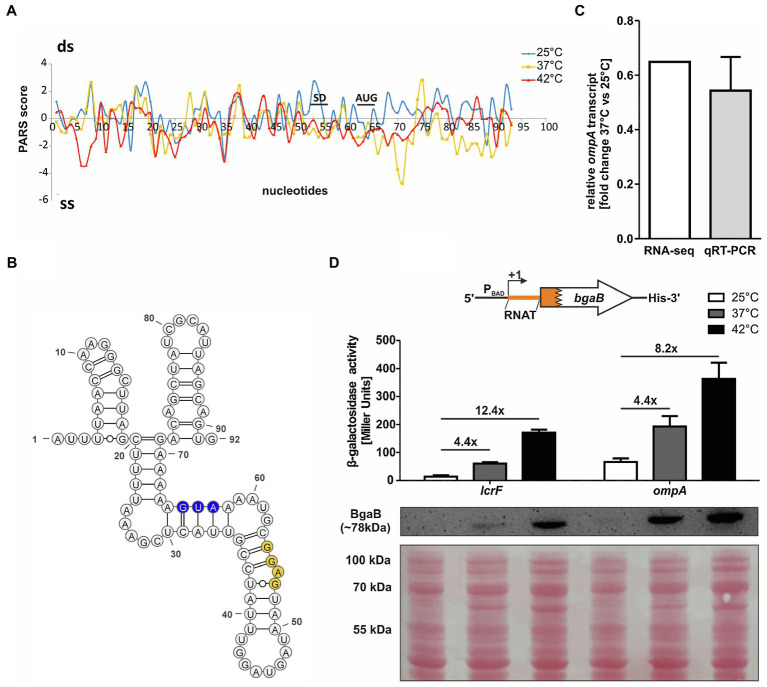
The *ompA* 5'-UTR contains a thermoresponsive RNA structure. **(A)** PARS profiles of the *ompA* RNAT (−62 nt to +30 nt from AUG) at 25, 37, and 42°C (Righetti et al., 2016). The potential SD sequence and the AUG are shown. A positive PARS score is indicative of a double-stranded (ds) conformation, whereas a negative PARS score suggests a single-stranded (ss) conformation. A drop from a positive score at 25°C to a negative score at 37°C suggests melting of a secondary structure. **(B)** PARS-derived secondary structure of the *ompA* RNAT at 25°C. The potential SD sequence is highlighted in yellow and its corresponding AUG in blue. –, AU pair; =, GC pair; and ○, GU pair. **(C)** Comparison of *ompA* transcript amount between 37 and 25°C by RNA sequencing (Nuss et al., 2015) and qRT-PCR (this study) of exponentially grown YPIII cells at 25 and 37°C, respectively. For qRT-PCR, the obtained data were normalized to *gyrB* and *nuoB*. qRT-PCR was prepared in biological triplicates and technical triplicates. **(D)** Translational control was measured by *bgaB* fusions. A schematic representation of the reporter gene fusion is displayed. The *ompA* RNAT was translationally fused to *bgaB* under control of the P_BAD_ promotor. As a control, the *lcrF* RNAT fusion was used. The fusion plasmids were introduced into *E. coli* cells and grown to an OD_600_ of 0.5 at 25°C. Subsequently, transcription was induced by the addition of 0.01% L-arabinose and the cultures were split and transferred into pre-warmed flask at 25, 37, and 42°C, respectively. After 30 min of incubation, samples were taken for β-galactosidase assays and Western blot analysis. Experiments were carried out in biological triplicates. Mean and corresponding standard deviations are shown. Western blot membranes were stained with Ponceau S as a loading control. One representative Western blot is shown. Analysis was carried out in biological triplicates.

A previously conducted global *in vitro* RNA structure probing analysis (Righetti et al., 2016) provided strong evidence for the existence of temperature-sensitive RNA structures in the 5'-UTRs of numerous mRNAs. One of the top candidates (rank #3 of chromosomally encoded mRNAs) discovered by this parallel analysis of RNA structures approach was the 5'-UTR of *ompA*. The RNA structure observed at 25°C was destabilized at higher temperatures (Figure 1A). This was especially prominent in the region around the SD sequence and in the early coding region. Here, the PARS profile dropped from positive to negative values indicative of a transition from a double-stranded (ds) to single-stranded (ss) conformation. This behavior is typical of zipper-like RNATs that facilitate ribosome binding at elevated temperatures (Kortmann and Narberhaus, 2012). The PARS analysis revealed an almost completely folded *ompA* 5'-UTR that is engaged in several stem-loop structures (Figure 1B). Strikingly, the SD sequence (53-GGAG-56) is imperfectly paired and contains a bulged adenine. It is very likely that this mismatched nucleotide in concert with four unpaired nucleotides (58-GUAA-61) between the SD sequence and the AUG start codon is key to the temperature sensitivity of this 5'-UTR.

To examine the regulation of *ompA* in detail, we first checked, whether transcription of the gene is temperature-controlled by performing qRT-PCR on total RNA samples from *Y. pseudotuberculosis* YPIII after growth at 25 and 37°C (Figure 1C). Consistent with already existing RNA-seq results (Nuss et al., 2015), we observed a fold change around 0.6 (37 vs. 25°C) showing a reduction in *ompA* mRNA at a higher temperature.

The RNA structurome data, in contrast, suggested an upregulation of OmpA protein at 37°C due to the thermoresponsive nature of the *ompA* 5'-UTR. To test this hypothesis, we translationally fused the *ompA* 5'-UTR to the *bgaB* gene coding for a heat-stable His-tagged β-galactosidase downstream of the arabinose-inducible P_BAD_ promotor and measured the β-galactosidase activity and protein amounts at 25°C and after a shift to 37 or 42°C. The well-studied *lcrF* RNAT of *Y. pseudotuberculosis* served as a positive control (Figure 1D). A clear increase in β-galactosidase activity and protein amount after the temperature shift supported the existence of a functional RNAT upstream of *ompA* able to confer translational repression at 25°C and induction at 37 or 42°C.

### A Stabilizing Point Mutation Prevents RNAT Regulation

**Figure 2:**
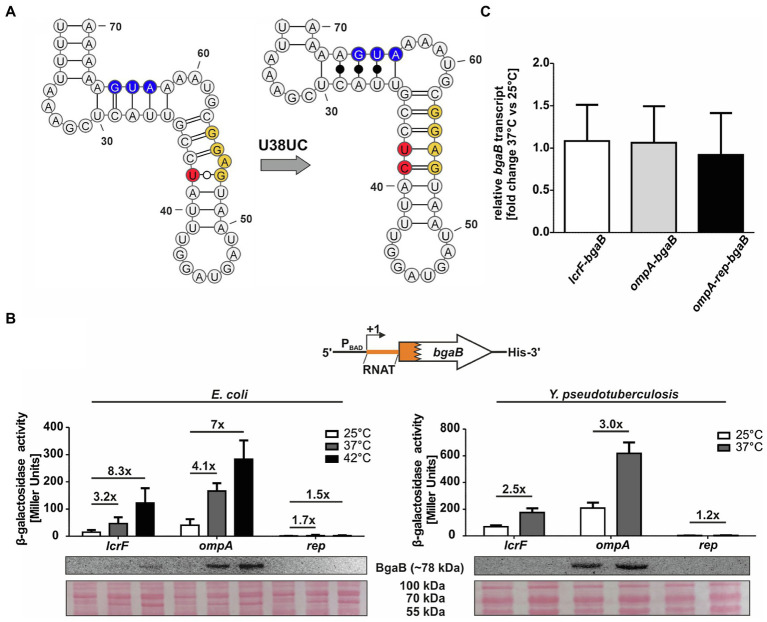
Mutation of the anti-SD sequence locks the RNAT in a closed conformation. **(A)** PARS-derived secondary structure of the *ompA* RNAT stem and the predicted stabilized structure at 25°C. The potential SD sequence is highlighted in yellow, the mutation site of the anti-SD sequence in red and the AUG in blue. **(B)** Translational control was measured by *bgaB* fusions. A schematic representation of the reporter gene fusion is displayed. The *ompA* RNAT was translationally fused to *bgaB* under control of the P_BAD_ promotor. Additionally, a point mutation (U38UC) was introduced to stabilize the RNAT (rep). As a control, the *lcrF* RNAT fusion was used. The fusion plasmids were introduced into *E. coli* and *Y. pseudotuberculosis* YPIII cells and grown to an OD_600_ of 0.5 at 25°C. Subsequently, transcription was induced by the addition of 0.01% for *E. coli* or 0.1% for *Y. pseudotuberculosis* L-arabinose and the cultures were split and transferred into pre-warmed flask at 25 and 37°C, respectively. After 30 min of incubation, samples were taken for β-galactosidase assays, Western blot analysis, and qRT-PCR. Experiments were carried out in biological triplicates. Mean and corresponding standard deviations are shown. Western blot membranes were stained with Ponceau S as a loading control. One representative Western blot is shown. Analysis was carried out in biological triplicates. **(C)** Comparison of *bgaB* transcript amount between 37 and 25°C by qRT-PCR of exponentially grown YPIII cells harboring the *ompA* RNAT-*bgaB* fusion plasmids at 25 and 37°C, respectively. For qRT-PCR, the obtained data were normalized to *gyrB* and *nuoB*. qRT-PCR was prepared in biological triplicates and technical triplicates.

One way of verifying the contribution of a temperature-sensitive RNA structure in translational control is the construction of a stabilized version. Based on the PARS-derived secondary structure, we introduced a cytosine between U38 and A39 (called U38UC or “rep” for repressed variant) in the anti-SD sequence leading to a perfectly paired SD sequence without any mismatches (Figure 2A). The increased thermodynamic stability should prevent the RNA structure from melting at higher temperatures and thereby abolish RNAT functionality. Indeed, reporter gene assays revealed the absence of β-galactosidase protein and activity at 25 and 37°C in *E. coli* as well as in *Y. pseudotuberculosis* (Figure 2B). Apparently, the introduction of just one additional base pair was sufficient to abolish the temperature regulation. To solidify our claim that the 5'-UTR regulates translation (and not transcription or mRNA degradation), we analyzed the *bgaB* mRNA levels in all three tested *Y. pseudotuberculosis* strains by qRT-PCR and found that the transcript levels were the same at 25 and 37°C (Figure 2C).

### The *ompA* RNAT Opens at Higher Temperatures Around the SD Region

**Figure 3:**
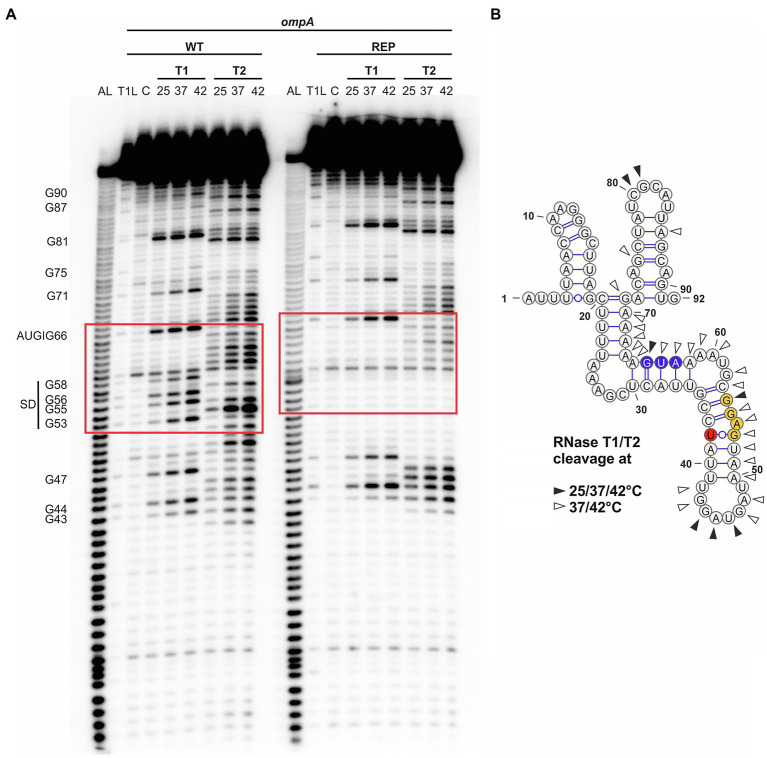
Accessibility of the ribosome-binding site of *ompA* is increased at higher temperatures. **(A)** Enzymatic structure probing of the *ompA* RNAT (WT) and its stabilized version (REP) at 25, 37, and 42°C. *In vitro* transcribed RNA was 5'-[^32^P] labeled and treated with RNases T1 and T2, respectively. AL, alkaline ladder; T1L, RNase T1 cleavage ladder in sequencing buffer at 37°C; C, RNA treated with water instead of RNases – cleavage control. Ribosome-binding site is highlighted by a red box. Experiment was carried out at least three times. **(B)** PARS-derived secondary structure of the *ompA* RNAT at 25°C. The potential SD sequence is highlighted in yellow, the mutation site of the anti-SD sequence in red, and the AUG in blue. Distinctive nucleotide cleavage is indicated by black and white arrows.

Next, we examined the structure and temperature-induced conformational changes in the RNAT by enzymatic RNA structure probing using *in vitro* transcribed and 5' end-labeled *ompA* RNA at 25, 37, and 42°C treated with RNases T1 (preferentially cleaves at unpaired guanines) and T2 (preferentially cleaves at unpaired adenines but also other ss residues; Figure 3A). The cleavage pattern supported the overall structure of the *ompA* 5'-UTR by prominent cleavage at 25°C in apical loops (e.g., C80 and G81). In accordance with the dynamic PARS-derived secondary structure (Figures 1A, 3B), residues around the SD sequence were sensitized against RNase cleavage at higher temperatures due to melting of the secondary structure. For example, nucleotides G53 and G55 were almost untouched by T1 at 25°C but efficiently cleaved at 37 and 42°C. Residual cleavage of the bulged A54 by T2 at 25°C massively increased at 37 and 42°C making it the most prominent cleavage product at high temperatures. Fully consistent with the reporter gene assays (Figure 2), the SD sequence of the rep variant was almost completely inaccessible to RNase cleavage demonstrating that melting of the RNAT is prevented by the point mutation, which locks the structure in a closed conformation.

### The Ribosome Gains Access to the *ompA* Transcript at 37°C

**Figure 4:**
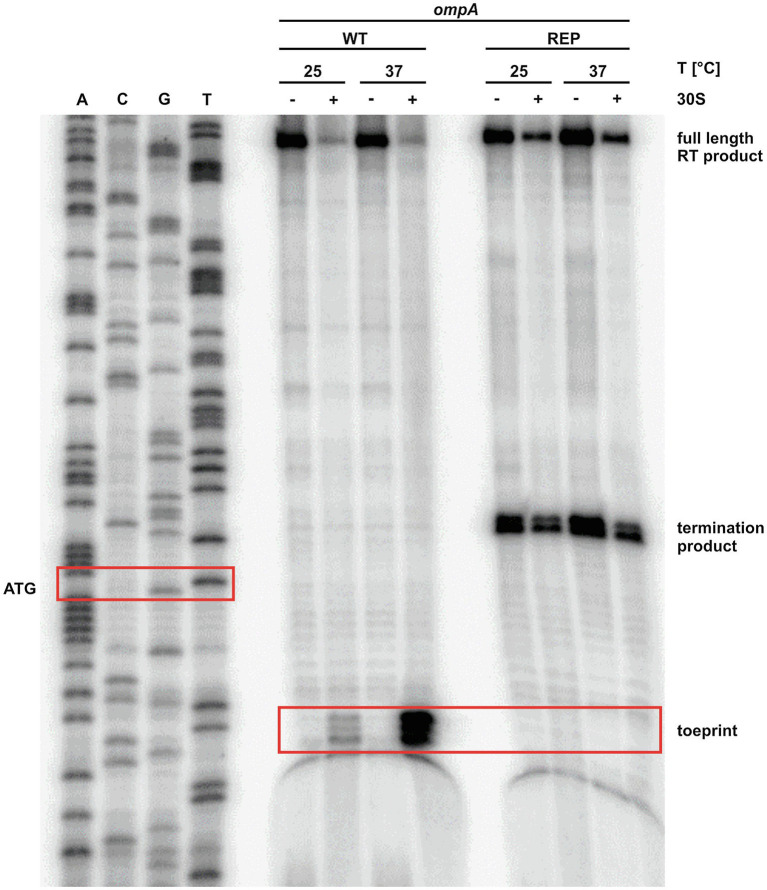
The ribosome binds to the *ompA* transcript at 37°C. Primer extension inhibition of the *ompA* RNAT (WT) and its stabilized version (REP) at 25 and 37°C with (+) and without (−) the addition of 30S ribosomal subunits. The signals represent the full-length reverse transcription (RT) product, the premature termination product, and the toeprint signal (caused by ribosome binding). ACGT lanes indicate sequencing reactions. Position of ATG and the toeprint signal is highlighted by red boxes. Experiment was carried out at least three times.

The observed melting of the *ompA* 5'-UTR at higher temperatures (Figures 1A, 3) together with the reporter gene studies (Figures 1D, 2) strongly suggested increased translation initiation at higher temperatures. We employed primer extension inhibition assays (toeprinting) to demonstrate ribosome binding. *In vitro* transcribed *ompA* RNA was reversely transcribed from a radiolabeled oligonucleotide in the presence or absence of 30S ribosomal subunits at 25 and 37°C. Ribosomes bound to the mRNA are stabilized in a ternary complex with tRNA^fMet^ and act as a roadblock for reverse transcription. This leads to premature termination of reverse transcription and thus shorter fragments (toeprints). As expected, in the presence of ribosomes, the toeprint signals in the appropriate distance from the AUG start codon were weak at 25°C but strong at 37°C (Figure 4). No toeprint signal, i.e., no ribosome binding, was found when the stabilized rep version was assayed. Instead, termination products independent of the presence of ribosomes appeared within the codon sequence, a phenomenon frequently observed downstream (considering the direction of the reverse transcriptase) of stable structures in stabilized RNATs (Böhme et al., 2012;^43,44^).

## Discussion

Sensing of and rapidly responding to the sudden external changes associated with host infection is crucial for enteric pathogens like *Y. pseudotuberculosis.* One parameter that predictably and reversibly changes during the transitions from the environment to a warm-blooded host and back into the environment is temperature. A fast and efficient way to reversibly modulate gene expression in response to temperature shifts is zipper-like RNATs because the mRNA is already available for translation (Grosso-Becera et al., 2015; Wei and Murphy, 2016; Loh et al., 2018). This is certainly the reason why RNATs are extremely widespread posttranscriptional control elements in numerous bacterial pathogens (Hoe and Goguen, 1993; Johansson et al., 2002; Böhme et al., 2012; Kouse et al., 2013; Loh et al., 2013; Weber et al., 2014; Wei et al., 2017; Twittenhoff et al., 2020a,b; Brewer et al., 2021).

Here, we describe the structural and functional features of an RNAT in the *Y. pseudotuberculosis ompA* transcript that was one of the prime candidates in our previous PARS study (Righetti et al., 2016). We were particularly interested in this candidate because a recent report demonstrated RNAT-controlled translation of *ompA* in *S. dysenteriae* (Murphy et al., 2020). Despite regulating the same gene, it is important to note that the *Yersinia* and *Shigella* RNATs are very different with respect to length, sequence, and structure. First, the *S. dysenteriae ompA* 5'-UTR is 133 nucleotides long, and the last 35 residues are sufficient to confer translational control. In contrast, the RNAT from *Y. pseudotuberculosis* is 92 nucleotides long and more densely folded. Second, the *S. dysenteriae* RNAT belongs to the fourU family showing the characteristic complementarity of the SD sequence to four consecutive uracil residues (Waldminghaus et al., 2007). The *Y. pseudotuberculosis* RNAT is not a member of this family. The inherent temperature lability is due to a bulged adenine residue in the center of the SD sequence, which pairs imperfectly with an anti-SD sequence comprised of CCUA. Introducing one additional residue that pairs with the exposed adenine completely eliminated the expression and heat induction even when sufficient *ompA* mRNA was present. This demonstrates the relevance of precisely balanced structural features in a functional RNAT to respond within the virulence-relevant temperature regime. Overall, the *ompA* thermometer is another interesting example of convergent evolution of RNATs as has previously been proposed for the regulation of CnfY-type toxins (Twittenhoff et al., 2020a b).

Many enterobacterial outer membrane protein genes are under tight posttranscriptional regulation (Guillier et al., 2006; Vogel and Papenfort, 2006). A highly conserved sRNA also believed to control *ompA* in *Yersinia* species is MicA (Udekwu et al., 2005). It binds across the SD sequence and prevents ribosome binding, which facilitates RNaseE-mediated cleavage. The temperature-induced structural transition of the *ompA* 5'-UTR from a closed to an open conformation potentially adds another layer of posttranscriptional control. Under circumstances when MicA is abundant, the sRNA might interfere with translation initiation. The exact interplay between the *ompA* thermosensor, MicA (and maybe other sRNAs), the RNA chaperone Hfq, and RNases is not yet understood. The situation, however, is somewhat reminiscent of the *Listeria prfA* thermometer that – in its open form – is able to interact with the two sRNAs, SreA and SreB, which decreases the level of PrfA and thereby links virulence gene expression to nutrient availability (Loh et al., 2009).

Having OmpA under temperature regulation supports its role as a virulence factor. There is growing evidence that the outer membrane protein is involved in many pathogenesis-related processes in diverse Gram-negative bacteria (Krishnan and Prasadarao, 2012). It is involved in adherence to epithelial cells (Smith et al., 2007; Gaddy et al., 2009), mouse mortality (Sánchez-Encinales et al., 2017), and biofilm formation (Weiser and Gotschlich, 1991; Azghani et al., 2002). OmpA of pathogenic *Yersinia* species is a highly immunogenic protein and may be an excellent vaccine candidate, due to its cross-immunogenicity and intense immune response among *Yersinia* species (Chen et al., 2015). Furthermore, *Y. pestis* and *Y. pseudotuberculosis ompA* mutants are defective in surviving in macrophages, and the *Y. pestis* mutant is outcompeted by the wild-type strain in a mouse-infection model (Bartra et al., 2012).

Given the importance of OmpA for efficient pathogenesis, it may be a promising target for treatment of infections caused by Gram-negative bacteria. A recently identified OmpA inhibitor, cyclic hexapeptide AOA-2, inhibits adherence to eukaryotic cells and biofilm formation (Vila-Farrés et al., 2017). The compound attenuates virulence by reducing the dissemination between organs and decreases mouse mortality after *A. baumannii*, *E. coli*, and *Pseudomonas aeruginosa* infections. In combination with other antimicrobial agents, OmpA inhibitors might be helpful for combating multidrug-resistant bacteria and thereby reducing mortality caused by infections with Gram-negative pathogens.

A recent study on OmpA in *Acinetobacter* sp. linked *ompA* expression to oxidative stress (Shahryari et al., 2021). The authors suggested a protective effect by the poor permeability of the slow OmpA porin compared to other outer membrane proteins, such as OmpC, which is known to produce larger pores, at least in *Salmonella* (van der Heijden et al., 2016). A connection between OmpA and oxidative stress is interesting since pathogens are typically exposed to a multitude of reactive oxygen species (ROS) upon infection of a host. An intriguing finding of our RNA structurome analyses is the presence of potential RNATs upstream of various oxidative stress response genes (Righetti et al., 2016;^43,44^). This is unlikely to be a coincidence and suggests parallel ways of host-body temperature-induced protective measures against ROS attack. Following up on RNAT-mediated control of genes combating ROS in pathogenic bacteria might be a worthwhile subject of future studies.

## Data Availability Statement

The raw data supporting the conclusions of this article will be made available by the authors, without undue reservation.

## Author Contributions

DS: conceptualization, data curation, formal analysis, investigation, validation, supervision, methodology, and writing original draft. CT: conceptualization, data curation, formal analysis, investigation, validation, supervision, and methodology. FB and MH: investigation, formal analysis, and methodology. FN: conceptualization, data curation, formal analysis, supervision, funding acquisition, project administration, writing original draft, review, and editing. All authors contributed to the article and approved the submitted version.

### Conflict of Interest

The authors declare that the research was conducted in the absence of any commercial or financial relationships that could be construed as a potential conflict of interest.

## References

[1] Ambrosi C. Pompili M. Scribano D. Zagaglia C. Ripa S. Nicoletti M. (2012). Outer membrane protein A (OmpA): a new player in *Shigella flexneri* protrusion formation and inter-cellular spreading. PLoS One 7:e49625. 10.1371/journal.pone.0049625, PMID: 23166731 PMC3498225

[2] Azghani A. O. Idell S. Bains M. Hancock R. E. (2002). *Pseudomonas aeruginosa* outer membrane protein F is an adhesin in bacterial binding to lung epithelial cells in culture. Microb. Pathog. 33, 109–114. 10.1006/mpat.2002.0514, PMID: 12220987

[3] Bartra S. S. Gong X. Lorica C. D. Jain C. Nair M. K. M. Schifferli D.. (2012). The outer membrane protein A (OmpA) of *Yersinia pestis* promotes intracellular survival and virulence in mice. Microb. Pathog. 52, 41–46. 10.1016/j.micpath.2011.09.009, PMID: 22023991 PMC3237948

[4] Böhme K. Steinmann R. Kortmann J. Seekircher S. Heroven A. K. Berger E.. (2012). Concerted actions of a thermo-labile regulator and a unique intergenic RNA thermosensor control *Yersinia* virulence. PLoS Pathog. 8:e1002518. 10.1371/journal.ppat.1002518, PMID: 22359501 PMC3280987

[5] Bölin I. Norlander L. Wolf-Watz H. (1982). Temperature-inducible outer membrane protein of *Yersinia pseudotuberculosis* and *Yersinia enterocolitica* is associated with the virulence plasmid. Infect. Immun. 37, 506–512. 10.1128/IAI.37.2.506-512.1982, PMID: 6749681 PMC347563

[6] Brantl S. Wagner E. G. (1994). Antisense RNA-mediated transcriptional attenuation occurs faster than stable antisense/target RNA pairing: an *in vitro* study of plasmid pIP501. EMBO J. 13, 3599–3607. 10.1002/j.1460-2075.1994.tb06667.x, PMID: 7520390 PMC395265

[7] Brewer S. M. Twittenhoff C. Kortmann J. Brubaker S. W. Honeycutt J. Massis L. M.. (2021). A *Salmonella* Typhi RNA thermosensor regulates virulence factors and innate immune evasion in response to host temperature. PLoS Pathog. 17:e1009345. 10.1371/journal.ppat.1009345, PMID: 33651854 PMC7954313

[8] Chen Y. Duan R. Li X. Li K. Liang J. Liu C.. (2015). Homology analysis and cross-immunogenicity of OmpA from pathogenic *Yersinia enterocolitica*, *Yersinia pseudotuberculosis* and *Yersinia pestis*. Mol. Immunol. 68, 290–299. 10.1016/j.molimm.2015.09.016, PMID: 26435220

[9] Choi C. H. Lee E. Y. Lee Y. C. Park T. I. Kim H. J. Hyun S. H.. (2005). Outer membrane protein 38 of *Acinetobacter baumannii* localizes to the mitochondria and induces apoptosis of epithelial cells. Cell. Microbiol. 7, 1127–1138. 10.1111/j.1462-5822.2005.00538.x, PMID: 16008580

[10] Clavel T. Germon P. Vianney A. Portalier R. Lazzaroni J. C. (1998). TolB protein of *Escherichia coli* K-12 interacts with the outer membrane peptidoglycan-associated proteins Pal, Lpp and OmpA. Mol. Microbiol. 29, 359–367. 10.1046/j.1365-2958.1998.00945.x 9701827

[11] Confer A. W. Ayalew S. (2013). The OmpA family of proteins: roles in bacterial pathogenesis and immunity. Vet. Microbiol. 163, 207–222. 10.1016/j.vetmic.2012.08.019, PMID: 22986056

[12] Douchin V. Bohn C. Bouloc P. (2006). Down-regulation of porins by a small RNA bypasses the essentiality of the regulated intramembrane proteolysis protease RseP in *Escherichia coli*. J. Biol. Chem. 281, 12253–12259. 10.1074/jbc.M600819200, PMID: 16513633

[13] Gaddy J. A. Tomaras A. P. Actis L. A. (2009). The *Acinetobacter baumannii* 19606 OmpA protein plays a role in biofilm formation on abiotic surfaces and in the interaction of this pathogen with eukaryotic cells. Infect. Immun. 77, 3150–3160. 10.1128/IAI.00096-09, PMID: 19470746 PMC2715673

[14] Gaubig L. C. Waldminghaus T. Narberhaus F. (2011). Multiple layers of control govern expression of the *Escherichia coli ibpAB* heat-shock operon. Microbiology 157, 66–76. 10.1099/mic.0.043802-0, PMID: 20864473

[15] Grosso-Becera M. V. Servin-González L. Soberón-Chávez G. (2015). RNA structures are involved in the thermoregulation of bacterial virulence-associated traits. Trends Microbiol. 23, 509–518. 10.1016/j.tim.2015.04.004, PMID: 25999019

[16] Guillier M. Gottesman S. Storz G. (2006). Modulating the outer membrane with small RNAs. Genes Dev. 20, 2338–2348. 10.1101/gad.1457506, PMID: 16951250

[17] Hanahan D. (1983). Studies on transformation of *Escherichia coli* with plasmids. J. Mol. Biol. 166, 557–580. 10.1016/S0022-2836(83)80284-8, PMID: 6345791

[18] Hartz D. McPheeters D. S. Traut R. Gold L. (1988). Extension inhibition analysis of translation initiation complexes. Methods Enzymol. 164, 419–425. 10.1016/s0076-6879(88)64058-4 2468068

[19] Hoe N. P. Goguen J. D. (1993). Temperature sensing in *Yersinia pestis*: translation of the LcrF activator protein is thermally regulated. J. Bacteriol. 175, 7901–7909. 10.1128/JB.175.24.7901-7909.1993, PMID: 7504666 PMC206968

[20] Johansson J. Mandin P. Renzoni A. Chiaruttini C. Springer M. Cossart P. (2002). An RNA thermosensor controls expression of virulence genes in *Listeria monocytogenes*. Cell 110, 551–561. 10.1016/S0092-8674(02)00905-4, PMID: 12230973

[21] Kim S. W. Choi C. H. Moon D. C. Jin J. S. Lee J. H. Shin J. H.. (2009). Serum resistance of *Acinetobacter baumannii* through the binding of factor H to outer membrane proteins. FEMS Microbiol. Lett. 301, 224–231. 10.1111/j.1574-6968.2009.01820.x, PMID: 19878322

[22] Knittel V. Vollmer I. Volk M. Dersch P. (2018). Discovering RNA-based regulatory systems for *Yersinia* virulence. Front. Cell. Infect. Microbiol. 8:378. 10.3389/fcimb.2018.00378 30460205 PMC6232918

[23] Kortmann J. Narberhaus F. (2012). Bacterial RNA thermometers: molecular zippers and switches. Nat. Rev. Microbiol. 10, 255–265. 10.1038/nrmicro2730, PMID: 22421878

[24] Kouse A. B. Righetti F. Kortmann J. Narberhaus F. Murphy E. R. (2013). RNA-mediated thermoregulation of iron-acquisition genes in *Shigella dysenteriae* and pathogenic *Escherichia coli*. PLoS One 8:e63781. 10.1371/journal.pone.0063781, PMID: 23704938 PMC3660397

[25] Krishnan S. Prasadarao N. V. (2012). Outer membrane protein A and OprF: versatile roles in gram-negative bacterial infections. FEBS J. 279, 919–931. 10.1111/j.1742-4658.2012.08482.x, PMID: 22240162 PMC3338869

[26] Loh E. Dussurget O. Gripenland J. Vaitkevicius K. Tiensuu T. Mandin P.. (2009). A trans-acting riboswitch controls expression of the virulence regulator PrfA in *Listeria monocytogenes*. Cell 139, 770–779. 10.1016/j.cell.2009.08.046 19914169

[27] Loh E. Kugelberg E. Tracy A. Zhang Q. Gollan B. Ewles H.. (2013). Temperature triggers immune evasion by *Neisseria meningitidis*. Nature 502, 237–240. 10.1038/nature12616, PMID: 24067614 PMC3836223

[28] Loh E. Righetti F. Eichner H. Twittenhoff C. Narberhaus F. (2018). RNA thermometers in bacterial pathogens. Microbiol. Spectr. 6. 10.1128/microbiolspec.RWR-0012-2017, PMID: 29623874 PMC11633587

[29] Mandin P. Johansson J. (2020). Feeling the heat at the millennium: thermosensors playing with fire. Mol. Microbiol. 113, 588–592. 10.1111/mmi.14468, PMID: 31971637

[30] Mizuno T. Chou M. Y. Inouye M. (1984). A unique mechanism regulating gene expression: translational inhibition by a complementary RNA transcript (micRNA). Proc. Natl. Acad. Sci. U. S. A. 81, 1966–1970. 10.1073/pnas.81.7.1966 6201848 PMC345417

[31] Murphy E. R. Roßmanith J. Sieg J. Fris M. E. Hussein H. Kouse A. B.. (2020). Regulation of OmpA translation and *Shigella dysenteriae* virulence by an RNA thermometer. Infect. Immun. 88:e00871–19. 10.1128/IAI.00871-19, PMID: 31792074 PMC7035939

[32] Nuss A. M. Heroven A. K. Waldmann B. Reinkensmeier J. Jarek M. Beckstette M.. (2015). Transcriptomic profiling of *Yersinia pseudotuberculosis* reveals reprogramming of the Crp regulon by temperature and uncovers Crp as a master regulator of small RNAs. PLoS Genet. 11:e1005087. 10.1371/journal.pgen.1005087, PMID: 25816203 PMC4376681

[33] Park J. S. Lee W. C. Yeo K. J. Ryu K. S. Kumarasiri M. Hesek D.. (2012). Mechanism of anchoring of OmpA protein to the cell wall peptidoglycan of the gram-negative bacterial outer membrane. FASEB J. 26, 219–228. 10.1096/fj.11-188425 21965596 PMC3250236

[34] Pfaffl M. W. (2001). A new mathematical model for relative quantification in real-time RT-PCR. Nucleic Acids Res. 29:e45. 10.1093/nar/29.9.e45, PMID: 11328886 PMC55695

[35] Prasadarao N. V. Blom A. M. Villoutreix B. O. Linsangan L. C. (2002). A novel interaction of outer membrane protein A with C4b binding protein mediates serum resistance of *Escherichia coli* K1. J. Immunol. 169, 6352–6360. 10.4049/jimmunol.169.11.6352, PMID: 12444142

[36] Prasadarao N. V. Wass C. A. Weiser J. N. Stins M. F. Huang S. H. Kim K. S. (1996). Outer membrane protein A of *Escherichia coli* contributes to invasion of brain microvascular endothelial cells. Infect. Immun. 64, 146–153. 10.1128/IAI.64.1.146-153.1996, PMID: 8557332 PMC173739

[37] Rasmussen A. A. Eriksen M. Gilany K. Udesen C. Franch T. Petersen C.. (2005). Regulation of *ompA* mRNA stability: the role of a small regulatory RNA in growth phase-dependent control. Mol. Microbiol. 58, 1421–1429. 10.1111/j.1365-2958.2005.04911.x, PMID: 16313626

[38] Righetti F. Nuss A. M. Twittenhoff C. Beele S. Urban K. Will S.. (2016). Temperature-responsive *in vitro* RNA structurome of *Yersinia pseudotuberculosis*. Proc. Natl. Acad. Sci. U. S. A. 113, 7237–7242. 10.1073/pnas.1523004113, PMID: 27298343 PMC4932938

[39] Sánchez-Encinales V. Álvarez-Marín R. Pachón-Ibáñez M. E. Fernández-Cuenca F. Pascual A.. (2017). Overproduction of outer membrane protein A by *Acinetobacter baumannii* as a risk factor for nosocomial pneumonia, bacteremia, and mortality rate increase. J. Infect. Dis. 215, 966–974. 10.1093/infdis/jix010, PMID: 28453834

[40] Shahryari S. Talaee M. Haghbeen K. Adrian L. Vali H. Shahbani Zahiri H.. (2021). New provisional function of OmpA from *Acinetobacter* sp. strain SA01 based on environmental challenges. mSystems 6:e01175–20. 10.1128/mSystems.01175-20, PMID: 33436517 PMC7901484

[41] Smith S. G. Mahon V. Lambert M. A. Fagan R. P. (2007). A molecular Swiss army knife: OmpA structure, function and expression. FEMS Microbiol. Lett. 273, 1–11. 10.1111/j.1574-6968.2007.00778.x, PMID: 17559395

[42] Steinmann R. Dersch P. (2013). Thermosensing to adjust bacterial virulence in a fluctuating environment. Future Microbiol. 8, 85–105. 10.2217/fmb.12.129, PMID: 23252495

[43] Twittenhoff C. Brandenburg V. B. Righetti F. Nuss A. M. Mosig A. Dersch P.. (2020a). Lead-seq: transcriptome-wide structure probing *in vivo* using lead(II) ions. Nucleic Acids Res. 48:e71. 10.1093/nar/gkaa404, PMID: 32463449 PMC7337928

[44] Twittenhoff C. Heroven A. K. Mühlen S. Dersch P. Narberhaus F. (2020b). An RNA thermometer dictates production of a secreted bacterial toxin. PLoS Pathog. 16:e1008184. 10.1371/journal.ppat.1008184, PMID: 31951643 PMC6992388

[45] Udekwu K. I. Darfeuille F. Vogel J. Reimegård J. Holmqvist E. Wagner E. G. (2005). Hfq-dependent regulation of OmpA synthesis is mediated by an antisense RNA. Genes Dev. 19, 2355–2366. 10.1101/gad.354405, PMID: 16204185 PMC1240044

[46] van der Heijden J. Reynolds L. A. Deng W. Mills A. Scholz R. Imami K.. (2016). *Salmonella* rapidly regulates membrane permeability to survive oxidative stress. MBio 7:e01238. 10.1128/mBio.01238-16, PMID: 27507830 PMC4992977

[47] Vila-Farrés X. Parra-Millán R. Sánchez-Encinales V. Varese M. Ayerbe-Algaba R. Bayó N.. (2017). Combating virulence of gram-negative bacilli by OmpA inhibition. Sci. Rep. 7:14683. 10.1038/s41598-017-14972-y, PMID: 29089624 PMC5666006

[48] Vogel J. Papenfort K. (2006). Small non-coding RNAs and the bacterial outer membrane. Curr. Opin. Microbiol. 9, 605–611. 10.1016/j.mib.2006.10.006, PMID: 17055775

[49] Waldminghaus T. Heidrich N. Brantl S. Narberhaus F. (2007). FourU: a novel type of RNA thermometer in *Salmonella*. Mol. Microbiol. 65, 413–424. 10.1111/j.1365-2958.2007.05794.x, PMID: 17630972

[50] Weber G. G. Kortmann J. Narberhaus F. Klose K. E. (2014). RNA thermometer controls temperature-dependent virulence factor expression in *Vibrio cholerae*. Proc. Natl. Acad. Sci. U. S. A. 111, 14241–14246. 10.1073/pnas.1411570111, PMID: 25228776 PMC4191814

[51] Wei Y. Kouse A. B. Murphy E. R. (2017). Transcriptional and posttranscriptional regulation of *Shigella shuT* in response to host-associated iron availability and temperature. Microbiology 6:e00442. 10.1002/mbo3.442, PMID: 28127899 PMC5458455

[52] Wei Y. Murphy E. R. (2016). “Temperature-dependent regulation of bacterial gene expression by RNA thermometers” in Nucleic Acids – From Basic Aspects to Laboratory Tools. eds. Larramendy M. Soloneski S. (London:IntechOpen).

[53] Weiser J. N. Gotschlich E. C. (1991). Outer membrane protein A (OmpA) contributes to serum resistance and pathogenicity of *Escherichia coli* K-1. Infect. Immun. 59, 2252–2258. 10.1128/IAI.59.7.2252-2258.1991, PMID: 1646768 PMC258003

[54] Yanisch-Perron C. Vieira J. Messing J. (1985). Improved M13 phage cloning vectors and host strains: nucleotide sequences of the M13mp18 and pUC19 vectors. Gene 33, 103–119. 10.1016/0378-1119(85)90120-9, PMID: 2985470

